# Biological Frailty Index in centenarians

**DOI:** 10.1007/s40520-021-01993-x

**Published:** 2021-10-16

**Authors:** Beatrice Arosio, Annalisa Geraci, Evelyn Ferri, Daniela Mari, Matteo Cesari

**Affiliations:** 1 Laboratorio di Geriatria, Department of Clinical Sciences and Community Health, University of Milan, Via Pace 9, 20122 Milan, Italy; 2 Geriatric Unit, Fondazione IRCCS Ca’ Granda Ospedale Maggiore Policlinico, Via Pace 9, 20122 Milan, Italy; 3 Laboratorio Sperimentale di Ricerche di Neuroendocrinologia Geriatrica ed Oncologica, Istituto Auxologico Italiano, IRCCS, Via Zucchi 18, 20095 Cusano Milanino, Italy; 4 Geriatric Unit, IRCCS Istituti Clinici Scientifici Maugeri, Via Camaldoli 64, 20138 Milan, Italy

**Keywords:** Centenarians, Biological frailty index, Biological reserves, Longevity

## Abstract

This study measured the subclinical frailty of centenarians by looking at the accumulation of their biological abnormalities. For this aim, a biological Frailty Index (FI) was computed in centenarians living in Northern Italy. The median value of the biological FI was 0.33 (interquartile range, IQR 0.28–0.41). The biological FI did not significantly differ between women (0.34, IQR 0.31–0.39) and men (0.32, IQR 0.26–0.43). The biological FI seems to have a narrower distribution compared to clinical FI we previously computed in the same cohort. In conclusion, our study suggests that centenarians benefit from exceptional biological reserves that might be underestimated by clinical appearances.

## Introduction

The amount of people reaching old age has been growing exponentially in the last decades, and centenarians represent the fastest-growing group (World Population Prospects 2019: Highlights; https://population.un.org/wpp/). Centenarians are persons with an extraordinary adaptive capacity, probably thanks to unusual functional reserves. Centenarians may live with debilitating disease, but still present an advantage in terms of incident disability and death [1, 2]**.**

They constitute a very heterogeneous population as result of lifestyle habits, environmental factors, and histories that have differently affected their biological and clinical profile over the life course [3, 4]. Thus, centenarians may be subjects with not only good but also very poor health status, as demonstrated by the different degrees of frailty we previously reported [5].

Aging occurs at molecular and cellular levels [6]. Interestingly, centenarians seem to express molecular signatures suggestive of a slower process compared to other persons [7, 8].

Recently, it has been explained that Frailty Index (FI) exclusively based on biological parameters may define the biological age of the individual, potentially capturing variations in the health status before the manifestation of clinical deficits [6, 9].

The aim of this study was to measure the subclinical frailty of centenarians by looking at the accumulation of their biological abnormalities. Since available measures of biological age are not optimized to disentangle the heterogeneity that characterizes centenarians [10], in this study, we have computed a biological FI by the means of blood tests in a cohort of well-characterized centenarians living in Northern Italy.

## Study design

The participants belonged to a large cohort enrolled during a study conducted between 2007 and 2014 and funded by the Italian Ministry of University and Scientific Research. The cohort was composed by 125 centenarians. Forty-six registry offices in Northern Italy were contacted to collect dates of birth of living people close to 100 years at the enrolment. Sixty-five out of 125 centenarians with all available variables needed for the computation of the biological FI were included. All these persons had a clinical FI already described [5].

Briefly, a trained multidisciplinary team went to each centenarian’s house or nursing home to administer a standard structured questionnaire and collect blood samples [11].

The biological FI was computed considering a total of 42 variables including routine blood tests [6], telomere length [12] and Apolipoprotein E genotype [9]. The 20th and 80th percentiles of each variable were considered as cut-points. The values under the 20th percentile and over the 80th percentile were considered abnormal. These biomarkers and their cut-points are presented in Table 1.

**Table 1:** Biomarkers and cut-points of the biological FI

Biomarkers	20th–80th percentile	
	Men	Women
Glycemia (mg/dl)	80–111	78–97
Insulin (µIU/ml)	2.8–11.8	3.6–12.0
Albumin (g/dl)	3.4–4.2	3.2–4.1
Urea (mg/dl)	39.8–74.6	37.2–71.0
Creatinine (mg/dl)	0.9–1.3	0.6–1.3
Uric Acid (mg/dl)	4.7–7.4	4.2–6.3
Cholesterol (mg/dl)	152–220	152–221
HDL (mg/dl)	41.0–62.0	39.8–59.0
Triglycerides (mg/dl)	78–154	73.4–149.6
Direct Bilirubin (mg/dl)	0.06–0.18	0.05–0.14
Total Bilirubin (mg/dl)	0.3–0.7	0.2–0.6
AST (U/L)	11–19	14–20
ALT (U/L)	5–10	5–13
GGT (U/L)	11.2–29.4	11.0–41.4
ALP (U/L)	65.0–133.4	58.4–135.6
Calcium (mg/dl)	9.5–10.2	9.4–10.3
Iron (µg/dl)	42.6–98.0	47.2–101.8
Phosphorus (mg/dL)	2.7–3.4	3.0–4.0
hs-CRP (mg/dl)	1.0–12.7	0.9–11.9
Lymphocytes (× 10^3^/µl)	1.14–1.78	1.06–1.86
Leukocytes (× 10^3^/µl)	5.8–7.8	5.2–7.4
Monocytes (× 10^3^/µl)	0.3–0.5	0.2–0.4
Haemoglobin (g/dl)	11.9–13.9	10.9–13.1
MCV (fl)	83–93	81–91
MCH (pg)	27.4–31.4	28.1–31.0
MCHC (g/dl)	32.6–35.6	33.1–36.1
Platelets (× 10^3^/µl)	175.0–254.0	162.4–296.2
CMV	Negativity	
PAI-1 Act (ng/ml)	1.0–5.1	1.0–3.9
Fibrinogen Antigen (mg/ml)	2.8–5.6	3.1–6.3
VWF Antigen (%)	165.6–294.4	186.0–318.4
Adamts-13 Antigen (%)	30.1–52.0	37.5–49.6
IGF-1 (ng/ml)	41.3–108.4	42.9–103.5
FT3 (pg/ml)	2.0–2.9	2.2–2.8
FT4 (ng/ml)	9.8–14.3	9.9–14.0
TSH (µIU/ml)	1.4–6.7	1.1–3.0
PTH (ng/l)	40.7–130.4	53.6–220.8
SHBG (nmol/l)	65.0–108.8	70.6–137.2
Testosteron (nmol/l)	4.7–15.4	0.2–0.9
25-OH Vitamin D (μg/l)	3.0–9.5	3.0–8.6
Telomere Length	> 0.76	> 0.87
Apolipoprotein E ε4	Negativity	

*HDL* High Density Lipoprotein, *AST* Aspartate Transaminase, *ALT* Alanine Transferase, *GGT* **γ**-Glutamyl Transpeptidase, *ALP* Alkaline Phosphatase, *hs-CRP* High Sensitivity C-reactive Protein, *MCV* Mean Corpuscolar Volume, *MCH* Mean Corpuscolar Hemoglobin, *MCHC* Mean Corpuscolar Hemoglobin Concentration, *CMV* Cytomegalovirus, *PAI-1 Act* Plasminogen Activator Inhibitor-1 Activity, *VWF* Von Willebrand Factor, *IGF-1* Insuline-like Growth Factor-1, *FT3* Free Triiodothyronine, *FT4* Free Thyroxine, *TSH* Thyroid-Stimulating Hormone, *PTH* Parathyroid Hormone, *SHBG* Sex Hormone Binding Globulin

Each biomarker was categorized to assume the value of 0 if its value fell within the range of normality or 1 if abnormal. The biological FI was then calculated as the ratio between the number of biomarkers presenting abnormal values and the number of considered biomarkers (*n *= 42).

## Results

Overall, a total of 65 centenarians (46 women and 19 men) were included in this study. The mean age of the sample was 101.3 (standard deviation, SD 2.0) years. The age was similar between women and men (101.2, SD 2.1 and 101.6, SD 2.0, respectively). As expected, the prevalence of women was higher than men (71% and 29%, respectively).

**Fig. 1:**
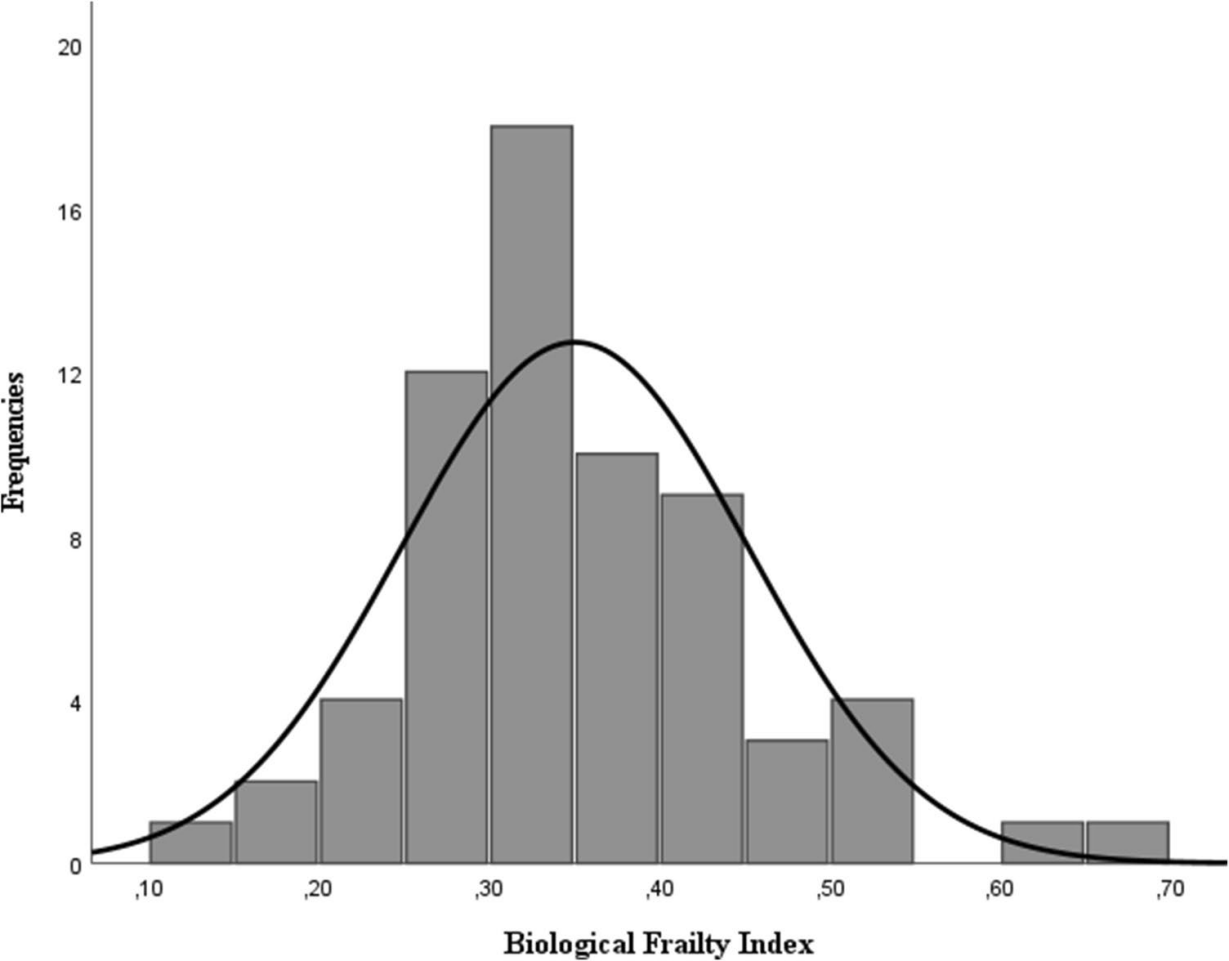
Distribution of the biological FI in centenarians

The median value of the biological FI was 0.33 (interquartile range, IQR 0.28–0.41). The biological FI did not significantly differ between women (0.34, IQR 0.31–0.39) and men (0.32, IQR 0.26–0.43). Figure 1 shows the distribution of the biological FI, which ranged between 0.11 and 0.69. Age was weakly correlated with the biological FI (Spearman’s r = 0.26, *p* = 0.04).

## Discussion

To our knowledge, this is the first study measuring a biological FI in a cohort of well-characterized centenarians. Interestingly, it seems to have a more narrow distribution compared to the clinical FI we previously computed [5].

In fact, in the same cohort, we reported a higher clinical FI (median 0.50, IQR 0.40–0.58), and a wider spectrum of values (ranging between 0.13 and 0.73) [5].

In a cohort of persons aged 80 years and older, it has been reported that the clinically fittest persons (FI values between 0 and 0.02) had a mean biological FI of 0.33, indicating that this latter is able to detect the subclinical accumulation of deficits and anticipate the clinical phenotype [9]. Similarly, community-dwelling men aged 40–79 showed a higher biological FI (based on routine blood tests) compared to the clinical one and a significant association with mortality and adverse health outcomes [6].

Nevertheless, our findings suggest that centenarians benefit from exceptional biological reserves that might be underestimated by clinical appearances. Indeed, in our cohort of centenarians, we got the counterintuitive finding of a biological FI lower than the clinical FI we previously reported.

This result may suggest that, at very advanced age, the biology of the system might be “better” than what clinically manifested. The hypothesis might be explained by the lower relevance that clinical constructs (e.g., definition of the diagnoses) may have with increasing age, especially if compared to the biological substratum feeding them [13]. After all, it is possible that several clinical deficits could be overestimated in centenarians. For example, some tools (e.g., Mini-Mental State Examination) are not validated for extremely old persons [14] and do not often consider peculiar characteristics (e.g., fatigue) potentially affecting their results.

We found a weak association between age and biological FI in centenarians probably because of the narrow range of chronological age and the similar biological FI observed in men and women. This last result is apparently in contrast with the so-called “sex-frailty paradox”, describing women as frailer than men but, at the same time, presenting longer life expectancy [15].

It is possible that, at an extremely advanced age (as in centenarians), the paradox may lose value because of the ceiling effect determined by the exceptional age and the favourable biology that allows it.

The main limitation of our study resides in the relatively low number of participants, which might have affected the statistical power of our analyses. We cannot also exclude that our sample does not represent the population of centenarians, and that third factors not considered in the study may differently explain our findings. For all these reasons, this study has to be considered an exploratory analysis that needs to be confirmed in a larger population.

In conclusion, our study suggests that centenarians benefit from exceptional biological reserves that might be underestimated by clinical appearances. Further studies are needed to disentangle the relationship between chronological age, biological age, and clinical complexity in older persons, especially at a very advanced age.

## Declarations

### Human and animal participants

All procedures involving human participants were in accordance with the ethical standards of the institutional and/or national research committee and with the 1964 Helsinki Declaration and its later amendments or comparable ethical standards.

## References

[1] Andersen SL (2020) Centenarians as Models of Resistance and Resilience to Alzheimer's Disease and Related Dementias. Adv Geriatr Med Res 2:e200018. 10.20900/agmr20200018 10.20900/agmr20200018 PMC7394313 32743561

[2] Andersen-Ranberg K, Schroll M, Jeune B (2001). Healthy centenarians do not exist, but autonomous centenarians do: a population-based study of morbidity among Danish centenarians. J Am Geriatr Soc.

[3] Ostan R, Monti D, Mari D, Arosio B, Gentilini D, Ferri E et al (2019) Heterogeneity of Thyroid Function and Impact of Peripheral Thyroxine Deiodination in Centenarians and Semi-Supercentenarians: Association With Functional Status and Mortality. J Gerontol A Biol Sci Med Sci 74:802–810. 10.1093/gerona/gly194 10.1093/gerona/gly194 30165411

[4] Salvioli S, Capri M, Bucci L, Lanni C, Racchi M, Uberti D et al (2009) Why do centenarians escape or postpone cancer? The role of IGF-1, inflammation and p53. Cancer Immunol Immunother 58:1909–1917. 10.1007/s00262-008-0639-6 10.1007/s00262-008-0639-6 PMC11030834 19139887

[5] Arosio B, Ferri E, Casati M, Mari D, Vitale G, Cesari M (2019) The Frailty Index in centenarians and their offspring. Aging Clin Exp Res 31:1685–1688. 10.1007/s40520-019-01283-7 10.1007/s40520-019-01283-7 31359370

[6] Blodgett JM, Theou O, Howlett SE, Rockwood K (2017) A frailty index from common clinical and laboratory tests predicts increased risk of death across the life course. Geroscience 39:447–455. 10.1007/s11357-017-9993-7 10.1007/s11357-017-9993-7 PMC5636769 28866737

[7] Tedone E, Arosio B, Gussago C, Casati M, Ferri E, Ogliari G et al (2014) Leukocyte telomere length and prevalence of age-related diseases in semisupercentenarians, centenarians and centenarians' offspring. Exp Gerontol 58:90–95. 10.1016/j.exger.2014.06.018 10.1016/j.exger.2014.06.018 24975295

[8] Sebastiani P, Federico A, Morris M, Gurinovich A, Tanaka T, Chandler KB et al (2021) Protein signatures of centenarians and their offspring suggest centenarians age slower than other humans. Aging Cell 20:e13290. 10.1111/acel.13290 10.1111/acel.13290 PMC7884029 33512769

[9] Mitnitski A, Collerton J, Martin-Ruiz C, Jagger C, von Zglinicki T, Rockwood K et al (2015) Age-related frailty and its association with biological markers of ageing. BMC Med 13:161. 10.1186/s12916-015-0400-x 10.1186/s12916-015-0400-x PMC4499935 26166298

[10] Bacalini MG, Gentilini D, Monti D et al (2021) No association between frailty index and epigenetic clocks in Italian semi-supercentenarians. Mech Ageing Dev 197:111514. 10.1016/j.mad.2021.111514 10.1016/j.mad.2021.111514 34098514

[11] Bucci L, Ostan R, Giampieri E, Cevenini E, Pini E, Scurti M et al (2014) Immune parameters identify Italian centenarians with a longer five-year survival independent of their health and functional status. Exp Gerontol 54:14–20. 10.1016/j.exger.2014.01.023 10.1016/j.exger.2014.01.023 24487345

[12] Tedone E, Arosio B, Gussago C et al (2014) Leukocyte telomere length and prevalence of age-related diseases in semisupercentenarians, centenarians and centenarians' offspring. Exp Gerontol 58:90–95. 10.1016/j.exger.2014.06.018 10.1016/j.exger.2014.06.018 24975295

[13] Cesari M, Perez-Zepeda MU, Marzetti E (2017) Frailty and Multimorbidity: Different Ways of Thinking About Geriatrics. J Am Med Dir Assoc 18:361–364. 10.1016/j.jamda.2016.12.086 10.1016/j.jamda.2016.12.086 28279606

[14] Arosio B, Ostan R, Mari D et al (2017) Cognitive status in the oldest old and centenarians: a condition crucial for quality of life methodologically difficult to assess. Mech Ageing Dev 165(Pt B):185–194. 10.1016/j.mad.2017.02.010 10.1016/j.mad.2017.02.010 28286214

[15] Gordon EH, Hubbard RE (2018)The Pathophysiology of Frailty: Why Sex Is So Important. J Am Med Dir Assoc 19:4–5. 10.1016/j.jamda.2017.10.009 10.1016/j.jamda.2017.10.009 29133018

